# Supplementary material for: Content-rich biological network constructed by mining PubMed abstracts
Source: BMC Bioinformatics. 2004 Oct 8;5:147. doi: 10.1186/1471-2105-5-147 (PMC528731; doi:10.1186/1471-2105-5-147)
Supplement: Additional File 2 — The original results of the above study (non-essential files are deleted to keep the file size under the limit set by BMC bioinformatics). [file 1471-2105-5-147-S2.bz2 › chilibotAdditionalFile2/dip10/201ID8652557E690/html/ERCC6_ERCC5.html]

 


 **ERCC6** and **ERCC5** 
  
Found 21 abstracts in PubMed, retrieved 10.  
 

 What does Google say? 
 PDF only 
| .edu only 

---

**Interactive relationship** (e.g. stimulation, inhibition, etc)

**Neutral relationship**- Here, we provide evidence that CSB  [ **ERCC6** ]  is found not only in the nucleoplasm but also in the nucleolus within a complex CSB  [ **ERCC6** ]  IP 150 that contains RNA pol I, TFIIH, and XPG  [ **ERCC5** ]  and promotes efficient rRNA synthesis.  Ref: 12419226 Mol Cell, 2002

**Non-interactive relationship** (e.g. studied together, co-existance, homology, etc.)

- In multivariate logistic regression analysis, low expression of ERCC1, XPB ERCC3, XPG  [ **ERCC5** ]   **ERCC5** , and CSB  [ **ERCC6** ]   **ERCC6**  was associated with a statistically significant increased risk for SCCHN.  Ref: 11900225 Cancer, 2002
- Clinically observed similarities between COFS syndrome and CS have been followed by discoveries of cases of COFS syndrome that are associated with mutations in the XPG  [ **ERCC5** ]  and CSB  [ **ERCC6** ]  genes.  Ref: 11443545 Am J Hum Genet, 2001
- CONCLUSIONS Reduced expression of ERCC1, XPB ERCC3, XPG  [ **ERCC5** ]   **ERCC5** , and CSB  [ **ERCC6** ]   **ERCC6**  is associated with a more than two fold increased risk of SCCHN.  Ref: 11900225 Cancer, 2002
- In contrast to cells bearing mutations in other components of the nucleotide excision repair apparatus XPB, XPD, XPG  [ **ERCC5** ]  and CSB  [ **ERCC6** ] , cells defective for the ERCC1 XPF structure specific nuclease are highly sensitive to cisplatin.  Ref: 12202770 Nucleic Acids Res, 2002
- Loss of heterozygosity LOH analysis for the XP, XPA, XPB, XPC, XPD, XPE, XPF, XPG  [ **ERCC5** ]  and the transcription coupled repair factor, Cockayne syndrome B CSB  [ **ERCC6** ]  revealed that NER factors were abnormal in 62.1 % of ovarian tumors 18 2 6.7% of colon 2 12 and 22.2% lung 2 9 carcinomas.  Ref: 11689286 Cancer Lett, 2001
- Loss of heterozygosity LOH analysis, using the microdissected tissues, for the XPA, XPB, XPC, XPD, XPE, XPF, XPG  [ **ERCC5** ]  and the transcription coupled repair factor, Cockayne syndrome B CSB  [ **ERCC6** ]  revealed that NER factors were abnormal in 30.0% 3 10 cases of oral squamous cell carcinomas.  Ref: 11496330 Oncol Rep< MedlineTA>Oncol Rep, 2001
- RESULTS The levels of ERCC1, XPB ERCC3, XPG  [ **ERCC5** ]   **ERCC5** , and CSB  [ **ERCC6** ]   **ERCC6**  transcripts were lower in cases than in controls.  Ref: 11900225 Cancer, 2002
